# Critical anatomic region of nasopalatine canal based on tridimensional analysis: cone beam computed tomography

**DOI:** 10.1038/srep12568

**Published:** 2015-08-06

**Authors:** Ana Fernández-Alonso, Juan Antonio Suárez-Quintanilla, Juan Muinelo-Lorenzo, Jesús Varela-Mallou, Ernesto Smyth Chamosa, María Mercedes Suárez-Cunqueiro

**Affiliations:** 1 Department of Stomatology, Medicine and Dentistry School, University of Santiago de Compostela, c/ Entrerrios, s/n, Santiago de Compostela, 15872, Spain; 2 Department of Anatomy, Medicine and Dentistry School, University of Santiago de Compostela, c/ San Francisco, s/n, Santiago de Compostela, 15782, Spain; 3 Department of Social Psychology, Basic Psychology and Methodology, Psychology School, University of Santiago de Compostela, c/ Xosé María Suárez Núñez, s/n, 15782, Santiago de Compostela, Spain; 4 Department of Preventive Medicine and Public Health, Medicine and Dentistry School, University of Santiago de Compostela, c/ San Francisco, s/n, Santiago de Compostela, 15782, Spain

## Abstract

The study aim of this was to define the critical anatomic region of the premaxilla by evaluating dimensions of nasopalatine canal, buccal bone plate (BBP) and palatal bone plate (PBP). 230 CBCTs were selected with both, one or no upper central incisors present (+/+, −/+, −/−) and periodontal condition was evaluated. T-student test, ANOVA, Pearson´s correlation and a multivariant-linear regression model (MLRM) were used. Regarding gender, significant differences at level 1 (lower NC) were found for: buccal-palatal, transversal and sagittal NC diameters, and NC length (NCL). Regarding dental status, significant differences were found for: total BBP length (tBL) and PBP width (PW2) at level 2 (NCL midpoint). NCL was correlated with PW2, tBL, and PBP length at level 3 (foramina of Stenson level). An MLRM had a high prediction value for NCL (69.3%). Gender is related to NC dimensions. Dental status has an influence on BBP dimensions, but does not influence on NC and PBP. Periodontal condition should be evaluated for precise premaxillae analysis NC diameters at the three anatomical planes are related to each other, while NCL is related to BBP and PBP lengths. A third of premaxilla is taken up by NC, thus, establishing the critical anatomic region.

The nasopalatine canal (NC), also called incisive canal or anterior palatine canal^1^,^2^, has been described as a canal located in the middle of the palate, just posterior to the roots of central maxillary incisors^1^. It has two openings: the incisive foramen, which is located underneath the incisive papilla, and the nasal opening or foramina of Stenson. The nasopalatine nerve, the terminal branch of descending nasopalatine artery, fibrous connective tissue, fat, and small salivary glands are the contents of the NC^3^.

High resorption rates in the premaxilla buccal to NC are commonly found in clinical practice. This resorption hinders ideal dental implant placement, and may give rise to aesthetic and functional problems^4^. In addition, an enlarged NC complicates osteotomy during implant surgery. Bone ridge resorption and nasopalatine canal morphology must be taken into consideration for adequate treatment planning in Dentistry^5^.

Nevertheless, no literature exists regarding the simultaneous three-dimensional analysis of the NC and adjacent bone. Determining the available bone in this anatomical region and the space occupied by the NC is challenging and has important clinical implications. It can help avoid complications resulting from injury of NC, such as neurovascular lesions and non-osseointegrated implants^6^. Although certain NC filling^7^,^8^,^9^ and nerve displacement techniques^10^ have been described, these are infrequently used and have limitations due to the variability of NC morphology with the possibility of multiple incisive foramen and foramina of Stenson, which our team described in an earlier study and which hinder neurovascular content displacement^5^.

The aim of this study was to define the critical anatomic region of the premaxilla by evaluating the dimensions of the nasopalatine canal (NC), the buccal bone plate (BBP) and the palatal bone plate (PBP) to canal.

## Material and Methods

Our overall sample consisted of 1551 consecutive CBCT of Spanish Caucasian patients referred to the Radiology Unit of the Medicine and Dentistry School at the University of Santiago de Compostela. These CBCTs were performed from July 2008 to March 2012 for treatment planning of various oral surgical procedures. A total of 230 CBCT were selected with both, one or no upper central incisors present (+/+,−/+, −/−) and were included in this study from March 2012 to December 2012. The CBCT selection method was judgment sampling^11^, because CBCT needed to fulfill certain inclusion and exclusion criteria. The sample size was large in order to be suitable for the statistical tests used.

### Inclusion criteria

The inclusion criteria were the following: 1) patients aged 18 years or older without defects in premaxilla area, 2) CBCT with voxel size of 0.3 mm or less. The exclusion criteria were: 1) impacted teeth in the anatomical area of interest, 2) presence of a radiolucent or radiopaque lesion, 3) root fragments present, 4) dental implants in the region of interest, 5) suspected NC pathology (cyst), 6) bone grafts, and 7) presence of orthodontic expanders.

### Ethics statement

Written informed consent was obtained from all patients. In compliance with ethical, methodologic and legal requirements, this study was approved by the Galician Ethics Committee of Clinical Research (Ref: 2012/272). The methods were performed in accordance with relevant guidelines and regulations.

### Image evaluation

CBCT were done using i-CAT® Model 17–19 (Imaging Sciences International, Inc., Hatfield, Pennsylvania, USA) with a flat-panel detector of amorphous silicon, and an exposure protocol of 120 kVp, a current of 5 mA, 14.7 s. The occlusal plane of patients was set parallel to the floor base by means of ear rods and a chin res. CBCT were evaluated by an experienced graduate student. DICOM files were reconstructed on computer (Samsung R522, Samsung Electronics, South Korea) using the 3D visualization and measurement software Carestream® CS 3D imaging software v3.2.12 (Carestream Health Inc., Rochester, NY, USA). Analysis was carried out using iCATVision software (i-CATVision 1.9, Imaging Sciences International, Inc., Hatfield, Pennsylvania, USA). The CBCT slice thickness was 0.25 mm.

### NC Measurements

The measurements were carried out at 3 different levels above the NC, the buccal bone plate (BBP) and palatal bone plate (PBP). Measurements are simultaneously taken for all three anatomical planes (axial, sagittal and coronal) as follows.

### Level 1

**Figure 1:**
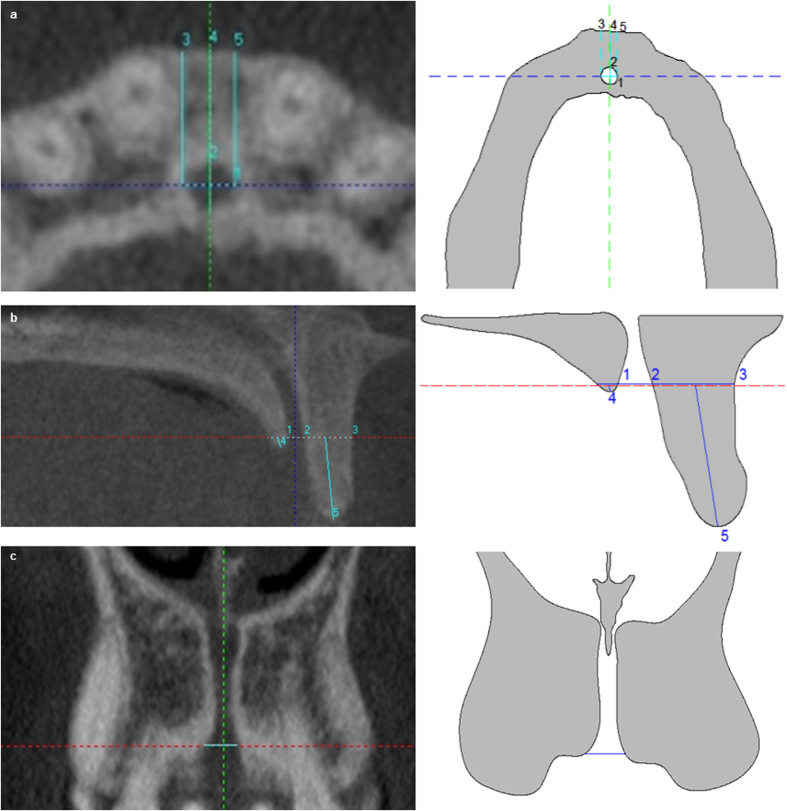
Radiographies and draw-scheme of level 1 show the follows: (**a**) Axial slice: (1) bpD1, (2) tD1, (3) LW1, (4) CW1 and (5) RW1. (**b**). Sagittal slice: (1) PW1, (2) SD1, (3) BW1, (4) PL1 and (5) BL1. (**c**) Coronal slice: CD1.

It is located based on the axial slice when the incisive foramen of NC is completely closed. In axial slice the following measurements were taken: (1) buccal-palatal diameter (bpD1) and transversal diameter (tD1) of NC. The bpD1 corresponded to maximum diameter between buccal and palatal bone cortical of NC; the tD1 is the maximum diameter perpendicular to bpD1; and, (2) BBP widths. The distance from BBP to buccal wall of NC at three points of NC: left (LW1), central (CW1) and right (RW1) (Fig. 1).

In coronal slice the following measurement was taken on the horizontal line: (1) NC diameter at level 1 (CD1) (Fig. 1).

In sagittal slice the following measurements are taken on the horizontal line: (1) BBP width at level 1 (BW1), (2) NC sagittal diameter at level 1(SD1), and (3) PBP width at level 1 (PW1). In addition, the following measurements are taken from the horizontal line to the inferior edges of BBP and PBP: (1) BBP length at level 1(BL1), and (2) PBP length at level 1 (PL1) (Fig. 1).

### Level 2

It is located by moving the horizontal line to the middle point of NC length (NCL) on the sagittal plane. NCL is defined as the distance from the incisive foramina to foramina of Stenson.

**Figure 2:**
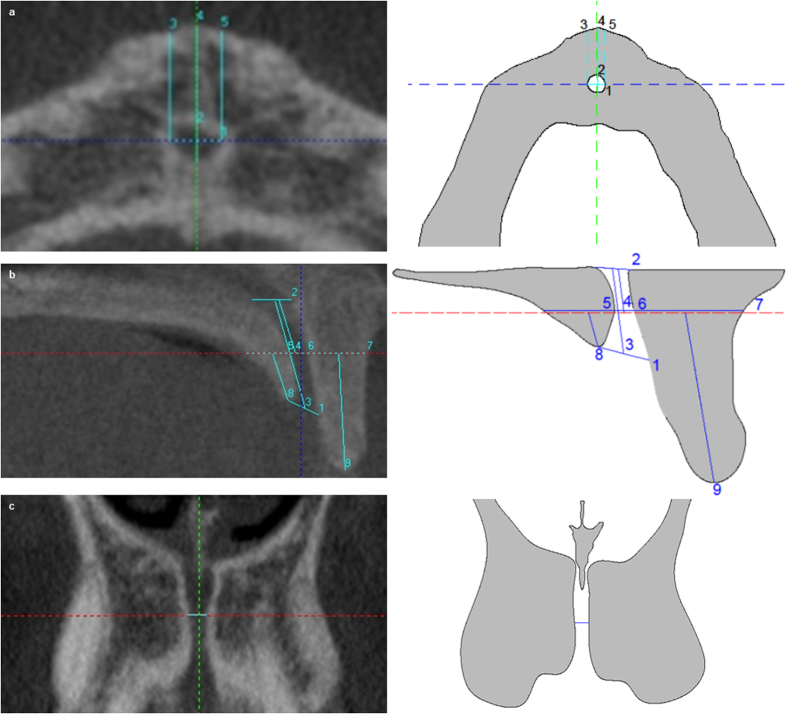
Radiographies and draw- scheme of level 2 show the follows: (**a**) Axial slice: (1) bpD2, (2) tD2, (3) LW2, (4) CW2 and (5) RW2. (**b**). Sagittal slice: (1) NC diameter in coronal region, (2) CD3, (3) NCL, (4) middle of NCL, (5) PW2, (6) SD2, (7) BW2, (8) PL2 and (9) BL2. (**c**) Coronal slice: CD2.

In axial slice the following measurements were taken: (1) buccal-palatal diameter (bpD2) and transversal diameter (tD2) of NC. The bpD2 corresponded to maximum diameter between buccal and palatal bone cortical of NC; the tD2 is the maximum diameter perpendicular to bpD2; and, (2) BBP widths. The distance from BBP to buccal wall of NC at three points of NC: left (LW2), central (CW2) and right (RW2) (Fig. 2).

In coronal the following measurement was taken on the horizontal line: (1) NC diameter at level 2 (CD2) (Fig. 2).

In sagittal slice the following measurements were taken on the horizontal line: (1) BBP width at level 2 (BW2), (2) NC sagittal diameter at level 2(SD2), and (3) PBP width at level 2 (PW2). In addition, the following measurements were taken from the horizontal line to the inferior edges of BBP and PBP: (1) BBP length at level 2 (BL2), and (2) PBP length at level 2 (PL2) (Fig. 2).

### Level 3

**Figure 3:**
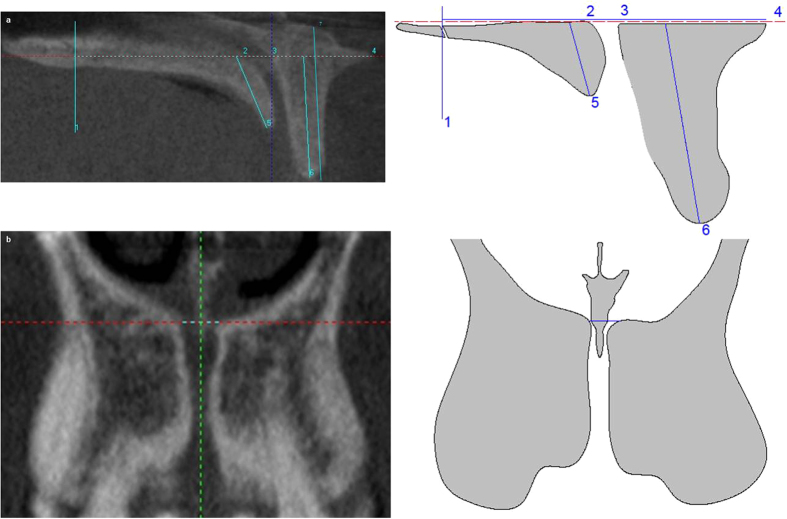
Radiographies and draw- scheme of level 3 show the follows: (**a**) Sagittal slice: (1) the most posterior point of the transverse palatine suture, (2) PW3, (3) SD3, (4) BW3, (5) PL3, (6) BL3 and (7) tBL. (**b**) Coronal slice: CD3.

It is located by moving the horizontal line to the foramina of Stenson on sagittal plane. In coronal slice the following measurement was taken on the horizontal line: (1) NC diameter at level 3 (CD3) (Fig. 3).

In sagittal slice the following measurements are taken on the horizontal line: (1) PBP width at level 3 (PW3), (2) the width from the most posterior point of the transverse palatine suture to the palatal wall of NC, (3) NC sagittal diameter at level 3 (SD3), and (4) PBP width at level 3 (PW3). In addition, the following measurements are taken from the horizontal line to the inferior edges of BBP and PBP: (1) BBP length at level 3 (BL3). (2) PBP length at level 3 (PL3), and, (3) the total BBP length (tBL) was also calculated by joining midpoints of coronal and apical BBP width (Fig. 3).

### Periodontal condition of the remaining teeth

Periodontal bone loss was evaluated on sagittal plane by measuring distance from the cemento-enamel junction to BBP in buccal surface and from the cemento-enamel junction to PBP in palate surface of teeth 11 and 21. Periodontal condition was defined as the mean value of these measurements for both teeth 11 and 21. Subjects were classified into three groups. The first group was normal periodontal condition ≤3 mm, and was based on the research by de Faria Vasconcelos *et al.*^12^. The other groups were added by our team to reflect different levels of periodontitis: moderate periodontitis (>3–≤6 mm) and severe periodontitis (>6 mm).

### Intraobserver agreement

One month later, the same observer assessed the NC measurements in 20 CBCTs to check the intraobserver variability, using the intraclass correlation coefficient for the NC measurements.

### Statistical analysis

All data were first analyzed using descriptive statistics. Differences in CBCT measurements were compared among subjects with and without teeth in the premaxila according to their dental status (−/+, +/+, −/−) by ANOVA with post hoc Bonferroni, Scheffe and Tukey-b tests; and according to gender by Student’s-t test. The proportion of NC diameter with respect to BBP in axial and sagittal slices was also evaluated. Pearson’s correlation was used to evaluate the association between measurements, periodontal condition and patient age. A multivariant linear regression model (MLRM) was used to predict the relation between measurements, dental status, and gender. The level of intraobserver agreement was assessed for anatomical measurements using the intraclass correlation coefficient. Statistical significance was set at p ≤ 0.05. Analyses were performed using SPSS 20.0 software for Windows (IBM SPSS Statistics, Chicago, IL, USA).

## Results

A total of 224 CBCT were included in the study. Six CBCTs (out of 230) were not included due to the following reasons: implant placed or bone grafting in the anterior maxilla, poor image quality, presence of maxillary expander, or a nasopalatine duct cyst. The study group comprised 108 males (48.2%) and 116 females (51.8%) with a mean age of 47.28 years (Table 1). Dental status was 81.7% for dentulous (+/+) patients, 11.6% for edentulous (−/−) and 6.7% for partially edentulous (+/−) patients.

### Measurements for three anatomical planes of NC

A summary of NC, BBP, and PBP characteristics can be seen in Table 1. With respect to gender, the following variables present a significantly greater mean for males (p ≤ 0.05): bpD1, tD1, BBP width at axial levels 1 and 2 (LW1/2, CW1/2, RW1/2), CD1, SD1, BW1/2, NCL, PW2/3, and PL2/3 (Table 2). With respect to dental status, the following variables present significant differences between each group: age, and a lower mean value for edentulous subjects: age, LW1, CW1, RW1, BW1, BL1, BL2, BL3, tBL (Table 3). In Table 4 we can see the correlations between NC dimensions in the three anatomical planes, total BBP length and BBP width at sagittal level 2. Age had a direct correlation with CD1, that is, canal diameter is larger in older subjects.

Regarding the effect of periodontal condition of the remaining central incisors in +/+ and +/− subjects, a total of 53% had normal periodontal condition with a mean value of 2.48 ± 0.31 mm, 41.4% had moderate periodontitis with a mean value of 3.86 ± 0.79 mm and 5.6% had severe periodontitis with a mean value of 8.00 ± 1.48 mm. The correlations between periodontal condition and BBP, PBP and NC dimensions were the followings: direct correlations were established with age (r^2^ = 0.349, p < 0.001), tD1 (r^2 ^= 0.139, p = 0.050), CD1 (r^2 ^= 0.160, p = 0.024), PL1 (r^2 ^= 0.306, p < 0.001) and tBL (r^2 ^= 0.159, p = 0.025); and an indirect correlation was established with BL1 (r^2 ^ = −0.264, p < 0.001).

Multiple linear regression models (MLRM) were obtained for variables as follows: (1) NCL can be explained by the variables PL3 (B = 0. 0.595), BL3 (B = 0.212) and tBL (B = 0.151) in 69.3% of cases (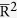 = 0.693; F = 169.16; p < 0.001), yielding the following MLRM: *NCL* = −*0.287* + *0.595PL3* + *0.212 BL3* + *0.151tBL*

(2) BW2 can be explained by the variables BW1 (B=0.794) in 62.8% of cases (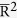
^ ^= 0.628; F = 377.97; p < 0.001), yielding the following MLRM: *BW2 = 1.95 + 0.794BW1*

(3) BpD1 can be explained by the variables tD1 (B = 0.603) in 36% of cases (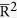 = 0.360; F = 126.55; p < 0.001), yielding the following MLRM: *BpD1* = *1.721* + *0.603tD1*

(4) tD2 can be explained by the variables bpD2 (B=0.632) and NCL (B = −0.158) in 43.10% of cases (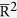 = 0.431; F = 85.36; p < 0.001), yielding the following MLRM: *tD2* = *2.158* + *0.632bpD2* – *0.158NCL*

(5) CD3 can be explained by the variables CD2 (B = 0.515) and SD3 (B = 0.289) in 42.6% of cases (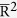 = 0.426; F = 83.78; p < 0.001), yielding the following MLRM: *CD3* = *1.284* + *0.515CD2* – *0.289SD3*

### Biological proportion between NC and BBP

The proportion of NC diameter with respect to BBP in axial and sagittal slices was also evaluated. In axial slice we found that bpD1 occupied an average percentage of 34.87 ± 9.34 (12.12–78.57) % and the bpD2 occupied 29.21 ± 9.36 (12.23–76.84) %. In the sagittal slice the SD1 occupied an average percentage of 31.40 ± 9.29 (9.52–75) %; SD2 a 22.88 ± 9.22 (7.69–76.98) %; and the SD3 25.62 ± 10.66 (4.84–76.19) %.

According to gender, the diameter proportion NC with respect to the BBP show the following percentages for males and females, respectively: bpD1 with 35.06 ± 9.07 (17.64–66.67) % and 34.68 ± 9.62 (12.12–78.57) %; bpD2 with 28.76 ± 8.91 (12.85–76.84) % and 29.63 ± 9.78 (12.23–75.00) %; SD1 with 31.47 ± 9.43 (9.52–75.00) % and 31.33 ± 9.19 (12.20–72.41) %; SD2 with 23.15 ± 10.82 (8.70–76.98) % and 22.62 ± 7.46 (7.69–40.98) %; and, SD3 with 25.62 ± 10.01 (9.93–59.17) % and 25.62 ± 11.27 (4.84–76.19) %. No significant differences between both genders were found.

With respect to dental status, the NC diameter proportion regarding BBP showed the following percentages for dentulous, edentulous and partially edentulous, respectively: bpD1 with 34.00 ± 8.36 (15.22–66.67) %, 40.59 ± 12.78 (17.64–78.57) % and 35.51 ± 10.89 (12.12–51.93) %, being significant different between +/+ and −/− p = 0.002; bpD2 with 28.94 ± 9.40 (12.23–76.84) %, 30.75 ± 10.20 (12.85–58.62) %, 29.81 ± 7.41 (15.63–40.54) %; SD1 with 30.80 ± 8.64 (9.52–75.00) %, 36.08 ± 11.90 (12.50–72.41) %, 30.59 ± 10.23 (13.24–48.65) %, being significantly different between +/+ and −/− p = 0.020; SD2 with 22.76 ± 9.46 (7.69–76.98) %, 24.41 ± 8.79 (10.81–45.16) %, 21.62 ± 6.84 (14.29–37.96) %; and SD3 with 25.61 ± 10.30 (4.84–61.11) %, 26.10 ± 14.39 (10.53–76.19) %, and 24.90 ± 7.73 (13.33–40.00) %.

### Intraobserver variability

The intraobserver variability was an intraclass correlation coefficient value ranging from 0.80 to 0.86 (95% confidence interval ranging from 0.57 to 0.94).

## Discussion

The use of three-dimensional images does not necessarily imply three-dimensional analysis. Unlike previous research based on CT or CBCT reconstructions^3^,^13^,^14^,^15^,^16^, the present study analyses NC and adjacent bone at the three anatomical planes simultaneously. 3D analysis of premaxilla dimensions is fundamental for determining adequate preoperative treatment. To the best of our knowledge, this is the first study to do so.

Our findings regarding axial NC diameters are in line with Song *et al.*^17^, who found transversal diameter to be greater than buccal-palatal diameter. Insofar as gender, significant differences were found for axial diameter only at level 1, but Thakur *et al.*^18^ found no such differences at any level in a study based on a hindu population. We found no differences regarding dental status, but this is not comparable with Mardinger *et al.*^13^ because these authors use the Lekholm and Zarb classification^19^. Regarding the correlation of transversal diameters, these were found to be directly proportional to each other at every level, with a moderate-strong correlation. In addition, they were proportional to sagittal and coronal diameters. There was an inverse correlation between BBP dimensions and axial diameters. Notably, the MLRM we obtained for predicting axial transversal diameter at level 2 had a predictive value of over 40%. Furthermore, it was the first time this analysis had been carried out.

With respect to sagittal NC diameters, we found significant differences according to gender at level 1, as did Tözüm *et al.*^20^. Other authors, such as Bornstein *et al.*^14^ and Thakur *et al.*^18^, found no such differences. However, we should point out that Bornstein *et al.*^14^ used the oblique measurement at this level instead of the horizontal measurement, resulting in greater values. Like other authors^14^,^20^, we found no differences with respect to dental status. This is in line with the centripetal resorption of maxillary bone theory^21^,^22^,^23^,^24^,^25^, indicating that anterior maxillary resorption occurs mainly in the buccal plate instead of the palatine plate.

Regarding coronal NC diameters, we obtained a similar mean value to Liang *et al.*^3^ (3.4 ± 0.9 mm) at level 1. The fact that these authors^3^ carried out their analysis on skulls is another confirmation of CBCT accuracy^26^,^27^,^28^,^29^,^30^. With respect to gender, we found male diameter to be significantly higher, but this is not supported by previous research. Insofar as dental status, our results were in line with Liang *et al.*^3^, who found no significant differences. Also like these authors^3^, we found a direct correlation with age; that is, diameter increased along with age.

With respect to NCL, the mean value we obtained is in line with other authors^14^,^20^. However, Liang *et al.*^3^ and Mraiwa *et al.*^16^ found a lower mean value. We should note that the sample size in the present study was considerably higher than the samples in those studies^3^,^16^. Regarding the influence of gender, like previous studies^14^,^17^,^18^,^20^,^31^, males presented significantly greater values. With respect to dental status, our results were nearly significant (p = 0.076), while previous studies^3^,^20^ reported significantly greater NCL in the dentulous group. Like other studies^13^,^17^, we found NCL in the dentulous group to be approximately 2-mm longer than the edentulous group, but these studies make no mention of significance. The present study found that NCL had a moderate to strong direct correlation with BBP and PBP length at levels 2 and 3 (BL2–3, PL2–3) as well as with BtL, while previous research did not analyze these correlations. Like Tözüm *et al.*^20^, we found no correlation between NCL and diameter. While other authors^14^,^20^ found an indirect correlation with age, we found no such correlation. Nevertheless, we did find that edentulous status increased with age (p < 0.001), thus suggesting a decrease in BBP length. In fact, when we applied an MLRM, we found that BBP length explained NCL with a predictive value of almost 70%.

To the best of our knowledge, this is the first study to analyze BBP in axial slice. This plane is the basis of CBCT, because coronal and sagittal planes are both determined in relation to axial slice^32^. We found that BBP width was significantly higher in males, and that it was higher in the coronal ridge region in the dentulous group, which is in line with others authors^23^,^24^,^25^, who explain that remodeling only occurs in coronal bone while basal bone is genetically determined. We found that axial BBP width was directly correlated with tBL and indirectly correlated with NC diameter in the three anatomic planes. We believe that this indirect correlation can be explained by a biological dimension adjustment, in other words, the proportion of bpD1/2 to CW1/2 remains constant. Thus, we have a + b = c, where a = bpD1/2 and b = CW1/2 and c is constant. Regardless of dental status, wider canals entail straighter ridges, and straighter canals entail wider ridges.

Like other authors^14^,^16^,^20^ we observed that sagittal BBP width increased to apical ridge and was greater in males^14^,^20^,^33^. As with the axial plane, only the coronal ridge region was significantly influenced by dental status, coinciding with other studies^13^,^14^,^20^. A previous study by our team^5^ did not find this difference because it only evaluated ridge width at one-third of the coronal BBP length. The present study demonstrates the importance of analyzing BBP ridge at different levels, because of its peculiar bone remodeling. We found sagittal width and length to be proportional, and Van der Weijden *et al.*^34^ reported that width presented more bone loss than length.

Regarding the BBP length, we found differences in dental status at all levels. Like Mardinger *et al.*^13^ and Tözüm *et al.*^20^, BBP length was significantly greater in the dentulous group. Moreover, the presence of only one central incisor (+−) also entailed a significantly greater value of BL2 and tBL. This is not supported by previous studies, nor is the direct correlation between BBP and PBP lengths.

In addition to dental status, the periodontal condition of remaining central incisors should be considered when the bone level and NC dimensions are evaluated. Several studies^12^,^35^,^36^ have demonstrated the accuracy of CBCT in the measurement of periodontal bone defects.

For higher quality images of periodontal structures, the voxel size should be inferior to 0.3 mm^29^ as in the present study. The mean age in the present study was similar to de Faria Vasconcelos *et al.*^12^, Grimard *et al.*^29^ and Mol *et al.*^37^, therefore, like these authors, we considered a measurement of higher than 3 mm between the cemento-enamel junction and the alveolar crest to indicate periodontitis.

Regarding PBP, width landmarks in the present study were not exactly the same as Tözüm *et al.*^20^, though both found a progressive increase and greater value for males in PBP width. Unlike these authors^20^, we found no significant differences regarding dental status. As occurred with BBP, PBP width and length were directly proportional. Insofar as PBP length, we obtained similar mean values for PL3 and differences for gender and dental status.

The critical anatomic region of premaxilla is defined by NC and BBP dimensions. We found that on average the NC diameter takes up approximately 1/3 of premaxilla. Although males presented greater NC and BBP dimensions in this area, both genders had similar biological dimension adjustment. Furthermore, we found that edentulous patients presented a higher proportion of NC diameter, entailing lower BBP width. The fact that bone resorption is related with tooth loss and mainly occurs at the coronal ridge helps to explain why we found a bone decrease of 6% at coronal ridge, 2% at middle ridge, and 1% at apical ridge.

With respect to limitations, the present study is based on the analysis of CBCT images, and patients gave their consent for this. Therefore, the present study had no other way of determining whether the subjects had had prior surgery.

## Conclusions

Gender is related to NC dimensions, BBP width and apical dimensions of PBP. Dental status has an influence on BBP dimensions, but does not influence on NC and PBP dimensions. Periodontal condition should be evaluated for a precise premaxillae analysis. NC diameters at the three anatomical planes are related to each other, while NCL is related to BBP and PBP lengths. BBP and PBP dimensions are proportional. A third of the premaxilla is taken up by the NC, thus, establishing the critical anatomic region.

## Additional Information

**How to cite this article**: Fernández-Alonso, A. *et al.* Critical anatomic region of nasopalatine canal based on tridimensional analysis: cone beam computed tomography. *Sci. Rep.* **5**, 12568; doi: 10.1038/srep12568 (2015).

## Figures and Tables

**Table 1:** Descriptive NC, BBP, and PBP measurements.

Levels	Parameters	Minimum (mm)	Maximum(mm)	Mean (mm)	SD (mm)
Level 1	bpD1	1.00	8.00	3.87	1.08
	tD1	1.00	9.75	3.91	1.18
	LW1	3.00	14.25	8.63	1.84
	CW1	1.50	11.25	7.32	1.61
	RW1	1.00	14.40	8.73	1.81
	CD1	1.00	9.75	3.60	1.24
	PW1	0.50	16.25	1.89	1.23
	SD1	1.00	7.25	3.38	1.06
	BW1	2.00	12.50	7.42	1.58
	BL1	0.56	16.66	9.04	2.76
	PL1	0.25	7.43	0.83	0.62
Level 2	NCL	4.35	23.57	12.34	2.79
	bpD2	1.25	7.50	3.26	0.98
	tD2	1.25	8.10	3.84	1.21
	LW2	2.40	14.75	8.29	1.83
	CW2	1.80	14.75	8.06	1.78
	RW2	1.80	14.50	8.48	1.94
	CD2	1.00	7.50	3.49	1.26
	PW2	2.40	21.75	6.65	2.53
	SD2	0.90	33.10	2.60	2.25
	BW2	2.25	14.75	8.53	1.76
	BL2	4.14	21.55	13.47	2.88
	PL2	2.26	16.40	5.55	1.87
Level 3	CD3	1.80	14.10	4.98	1.79
	PW3	8.50	31.75	22.32	3.61
	SD3	0.75	9.90	4.03	1.83
	BW3	2.25	18.00	11.83	2.94
	BL3	5.30	28.80	19.42	3.53
	PL3	4.60	23.95	11.93	2.84
	tBL	7.25	29.80	20.87	3.68

SD standard deviation.

**Table 2:** Effect of gender on NC BBP, and PBP measurements.

Parameters	Gender	N	Mean (mm)	SD (mm)	F	p
bpD1	Male	108	4.17	1.12	2.24	<0.001
	Female	116	3.60	0.97		
tD1	Male	108	4.06	1.32	4.56	0.05
	Female	116	3.76	1.02		
LW1	Male	108	9.30	1.74	0.73	<0.001
	Female	116	8.01	1.71		
CW1	Male	108	7.80	1.61	2.37	<0.001
	Female	116	6.88	1.49		
RW1	Male	108	9.42	1.74	1.54	<0.001
	Female	116	8.09	1.64		
CD1	Male	108	3.79	1.38	2.92	0.024
	Female	116	3.42	1.06		
SD1	Male	108	3.62	1.08	0.18	<0.001
	Female	116	3.15	1.00		
BW1	Male	108	7.96	1.63	4.35	<0.001
	Female	116	6.93	1.35		
NCL	Male	108	13.16	2.72	0.83	<0.001
	Female	116	11.58	2.64		
LW2	Male	108	8.77	1.82	0.19	<0.001
	Female	116	7.84	1.73		
CW2	Male	108	8.51	1.78	0.21	<0.001
	Female	116	7.63	1.69		
RW2	Male	108	8.99	1.97	1.72	<0.001
	Female	116	8.00	1.78		
PW2	Male	108	7.13	2.32	0.48	0.005
	Female	116	6.19	2.63		
BW2	Male	108	8.99	1.88	1.75	<0.001
	Female	116	8.11	1.53		
PL2	Male	108	5.83	1.72	0.00	0.033
	Female	116	5.29	1.98		
PW3	Male	108	23.14	3.59	0.90	0.001
	Female	116	21.56	3.48		
PL3	Male	108	12.80	3.02	3.31	<0.001
	Female	116	11.11	2.39		

SD standard deviation.

Statistically significant differences for p-values < 0.05.

**Table 3:** Effect of dental status on BBP, PBP and NC measurements.

Parameters	Dental status	N	Mean (mm)	SD (mm)	Minimum (mm)	Maximum (mm)	F	p-value of ANOVA	p-value of Bonferroni test
Age	+/+^a^	183	45.41	15.76	18.00	82.00	7.956	<0.001	0.001^a^
	−/−a	26	56.62	10.93	37.00	84.00			
	+/−	15	53.87	9.28	35.00	66.00			
LW1	+/+^a^	183	8.85	1.75	3.00	14.25	9.438	<0.001	0.001^a^
	−/−a	26	7.26	1.93	3.00	11.01			
	+/−	15	8.34	1.74	5.10	12.50			
CW1	+/+^a^	183	7.49	1.51	3.01	11.25	7.168	0.001	0.001^a^
	−/−a	26	6.27	1.88	1.50	9.90			
	+/−	15	7.09	1.65	3.61	9.76			
RW1	+/+^a^	183	8.96	1.70	4.25	14.40	12.291	<0.001	<0.001^a^
	−/−a	26	7.17	2.07	1.00	11.50			
	+/−	15	8.67	1.28	6.90	12.00			
BW1	+/+^a^	183	7.55	1.52	2.00	12.50	4.329	0.014	0.011^a^
	−/−a	26	6.60	1.78	2.00	10.50			
	+/−	15	7.32	1.51	3.60	9.30			
BL1	+/+^a^	183	9.47	2.55	0.56	16.66	15.596	<0.001	<0.001^a^
	−/−a	26	6.56	2.91	0.90	12.17			
	+/−	15	8.01	2.59	3.06	12.71			
PW2	+/+^a^	183	6.43	2.24	2.40	17.70	3.671	0.027	0.082^a^
	−/−a	26	7.60	3.58	3.50	21.75			
	+/−	15	7.61	3.22	4.20	13.50			
BL2	−/−b	26	10.64	3.69	4.14	20.01	16.722	<0.001	<0.001^a^ 0.010^b^
	+/+^a^	183	13.90	2.51	5.44	21.55			
	+/−b	15	13.24	2.86	7.69	18.12			
BL3	+/+^a^	183	19.88	3.09	11.95	28.80	11.847	<0.001	<0.001^a^
	−/−a	26	16.46	4.915	5.30	25.94			
	+/−	15	18.94	3.47	12.92	24.62			
tBL	+/+^a^	183	21.34	3.35	12.69	29.80	15.143	<0.001	<0.001^a^ 0.002^b^
	−/−b	26	17.36	4.32	7.25	25.65			
	+/−b	15	21.26	3.15	17.06	27.35			

+/+, Dentulous group; −/−, edentulous group; and +/−, partially edentulous group.

SD standard deviation.

Statistically significant differences for p-values < 0.05.

^a,b^For every variable, a letter indicates the group pair that was significantly difference.

**Table 4:** Correlations among NC measurements.

Parameters	Direct	p	Pearson’s Correlation (r^2^) Indirect	p
BpD1	tD1 (0.603)	<0.001	CW1 (−0.236);	<0.001
	CD1 (0.619)	<0.001	BW1 (−0.138)	0.039
	PW1 (0.155)	0.20	BL2 (−0.173)	0.009
	SD1 (0.831)	<0.001	BW3 (−0.136)	0.042
	bpD2(0.495)	<0.001		
	tD2 (0. 396)	<0.001		
	CD2 (0.432)	<0.001		
	PW2 (0.273)	<0.001		
	SD2 (0.336)	<0.001		
	CD3 (0.263)	<0.001		
	SD3 (0.197)	0.003		
tD1	CD1 (0.848)	<0.001	CW1 (−0.216)	0.001
	PW1 (0.136)	0.042	BW1 (−0.202)	0.002
	SD1 (0.573)	<0.001	LW2 (−0.145)	0.030
	bpD2 (0.441)	<0.001	CW2 (−0.226)	0.001
	tD2 (0.595)	<0.001	BW2 (−0.202)	0.002
	CD2 (0.654)	<0.001	BL2 (−0.202)	0.002
	SD2 (0.226)	0.001	BW3 (−0.144)	0.031
	CD3 (0.396)	<0.001	BL3 (−0.161)	0.016
	SD3 (0.227)	0.001	PL3 (−0.158)	0.018
CD1	age (0.182)	0.006	BW1 (−0.219)	0.001
	SD1 (0.610)	<0.001	LW2 (−0.160)	0.017
	bpD2 (0.437)	<0.001	CW2 (−0.219)	0.001
	tD2 (0.632)	<0.001	RW2 (−0.132)	0.049
	CD2 (0.653)	<0.001	BW2 (−0.188)	0.005
	SD2 (0.221)	0.001	BW3 (−0.142)	0.034
	CD3 (0.408)	<0.001		
	SD3 (0.260)	<0.001		
SD1	bpD2 (0.459)	<0.001	BW1 (−0.168)	0.012
	tD2 (0.371)	<0.001	PL1 (−0.168)	0.012
	CD2 (0.465)	<0.001	CW2 (-0.138)	0.039
	PW2 (0.256)	<0.001	BL2 (−0.166)	0.013
	SD2 (0.345)	<0.001	BW3 (−0.136)	0.041
	CD3 (0.264)	<0.001		
	SD3 (0.240)	<0.001		
NCL	PW2 (0.193)	0.004	tD2 (−0.194)	0.004
	BL2 (0.375)	<0.001	CD2(−0.171)	0.010
	PL2 (0.545)	<0.001		
	BW3 (0.180)	0.007		
	BL3 (0.660)	<0.001		
	PL3 (0.784)	<0.001		
	tBL (0.603)	<0.001		
bpD2	tD2 (0.641)	<0.001	LW2 (−0.134)	0.046
	CD2 (0.467)	<0.001	CW2 (−0.322)	<0.001
	SD2 (0.299)	<0.001	BW2 (−0.177)	0.008
	CD3 (0.275)	<0.001	BW3 (−0.194)	0.003
	SD3 (0.219)	0.001		
tD2	CD2 (0.737)	<0.001	CW2 (−0.146)	0.029
	SD2 (0.219)	0.001	RW2 (−0.153)	0.022
	CD3 (0.480)	<0.001	PL2 (−0.154)	0.021
	SD3 (0.282)	<0.001	PW3 (−0.135)	0.043
			BW3 (−0.184)	0.006
			PL3 (−0.162)	0.015
CD2	SD2 (0.219)	0.001	PL2 (−0.211)	0.001
	CD3 (0.595)	<0.001	BW3 (−0.180)	0.007
	SD3 (0.278)	<0.001	PL3 (−0.234)	<0.001
PW2	SD2 (0.271)	<0.001		
	PL2 (0.302)	<0.001		
	PL3 (0.332)	<0.001		
BW2	BL2 (0.162)	0.015	PW3 (−0.136)	0.041
	BW3 (0.443)	<0.001		
	BL3 (0.162)	0.015		
	tBL (0.156)	0.019		
CD3	SD3 (0.432)	<0.001	PW3 (−0.172)	0.010

Statistically significant differences p < 0.05.
